# Identification of transglutaminase 2 mediated polyaminated proteins in a hepatocellular cancer cell line

**DOI:** 10.1016/j.csbj.2025.10.009

**Published:** 2025-10-09

**Authors:** Don Benjamin, Danilo Ritz, Sunil Shetty, Sujin Park, Michael N. Hall

**Affiliations:** Biozentrum, University of Basel, Spitalstrasse 41, Basel 4056, Switzerland

**Keywords:** Polyamines, Putrescine, Spermine, Spermidine, Transglutaminase, TGM2, Polyamination

## Abstract

Polyamines are abundant metabolites that are involved in many cellular processes. Despite playing wide-ranging and essential roles in the cell, only a few examples of a specific polyamine function are known. Polyamination is the post-translational modification of a protein by polyamines (putrescine, spermidine or spermine). This reaction is catalyzed by transglutaminases (primarily TGM2) via a transamidation reaction that conjugates a polyamine to an acceptor glutamine in a target protein. Protein polyamination is poorly characterized due to technical challenges in detecting the polyaminated adduct, and is neglected in most proteomic surveys. We performed polyamination reactions using whole cell lysates from a mouse liver cancer cell line with elevated TGM2 expression. Two differently tagged polyamine analogs (Biotin-pentylamine and DNP-pentylamine) with distinct molecular masses were used, and the respective modified peptides were identified by mass spectrometry. 51 protein targets modified on 66 different sites were identified, in some cases with both donor polyamines. Many of the targets are involved in translation or cytoskeletal organization, and implicated in cancer.

## Graphical Abstract

**Figure:**
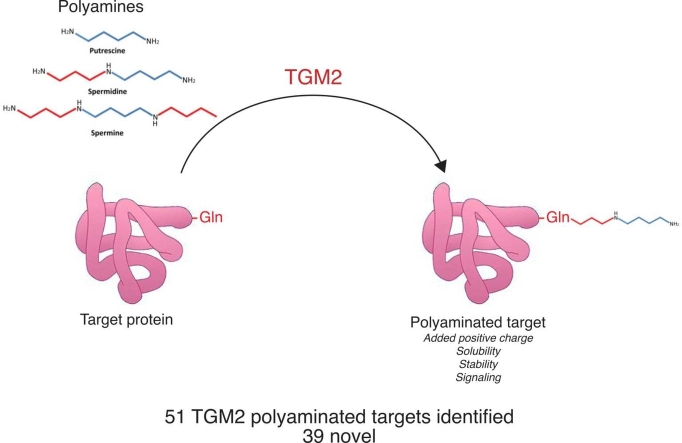

## 1 Introduction

Polyamines are an essential metabolite for cellular health, DNA replication, gene expression, protein translation and cell proliferation [1]. The major polyamine species (putrescine, spermidine and spermine), are produced endogenously as an off-shoot of arginine metabolism. Cadaverine, a product of gut bacterial metabolism, is also present in the serum in appreciable amounts. Despite having such wide-ranging effects, only a few specific functional roles are known for the polyamines, and most cellular effects of polyamines are attributed to their net positive charge. The best characterized specific role is for spermidine which is a precursor for the formation of hypusine on eIF5A [2]. Nonetheless, post-translational modification (PTM) of proteins by polyamination at glutamine residues has long been known [3], and in a few cases, the consequences of this modification has been elucidated in some detail. The dermonecrotizing toxin from *Bordetella* polyaminates host cell Rho at Q63 (and on analogous sites on Rac1 and Cdc42) to elicit pathogenic toxicity [4]. Mostly however, protein polyamination is performed by endogenous transglutaminases (TGM), a large family of proteins of which the most widely expressed and best studied is Transglutaminase 2 (TGM2). RhoA polyaminated by TGM2 has been shown to be constitutively activated [5], [6]. TGM2 polyaminated phospholipase A2 is also hyperactivated [7]. TGM2 polyamination stabilizes tubulin [8], while polyaminated lipocalin 2 is rapidly cleared [9]. Recently, the 4EBP proteins were shown to be polyaminated by TGM2 under hypoxic conditions to favor selective mRNA translation [10]. TGM2 also polyaminates BAF250a to modulate expression of specific genes [11]. Thus, there are examples of polyamines exerting an effect not merely due to mass action as abundant positively charged molecules (they are typically present at millimolar concentrations), but in a highly specific manner as a form of post-translational modification.

Proteomics of polyaminated proteins has lagged behind characterization of other PTMs due to the incompatibility of the polyaminated adduct with standard MS methodology, resulting either in its loss during sample purification, or destruction during data acquisition. The most recent proteomic study used an anti-spermine antibody to pull down in vitro polyaminated proteins from cell lysates and identified 254 polyaminated sites from 233 proteins [12]. Apart from this, however, there is an absence of large-scale surveys and most described examples have come via a bottom-up approach from investigation of individual proteins. In most of these studies, except for rare exceptions, the identity of the polyaminated glutamine residue is not known.

A mouse liver cancer cell line [13] that we developed depends exclusively on TGM2 for protein polyamination. Using two differently tagged polyamine probes, we identified 51 target proteins (66 sites). There is substantial overlap between the sites labelled using the different probes, giving confidence to the approach. Some of the hits have been previously reported but we also report a significant number of novel hits. In particular, we could identify the glutamine acceptor sites for all of the hits, something missing in most previous reports. Among the targets are proteins involved in translation, cytoskeletal organization and cancer.

## 2 Results

### 2.1 Characterization of CB1 as exclusively dependent on TGM2 for polyamination

**Fig. 1:**
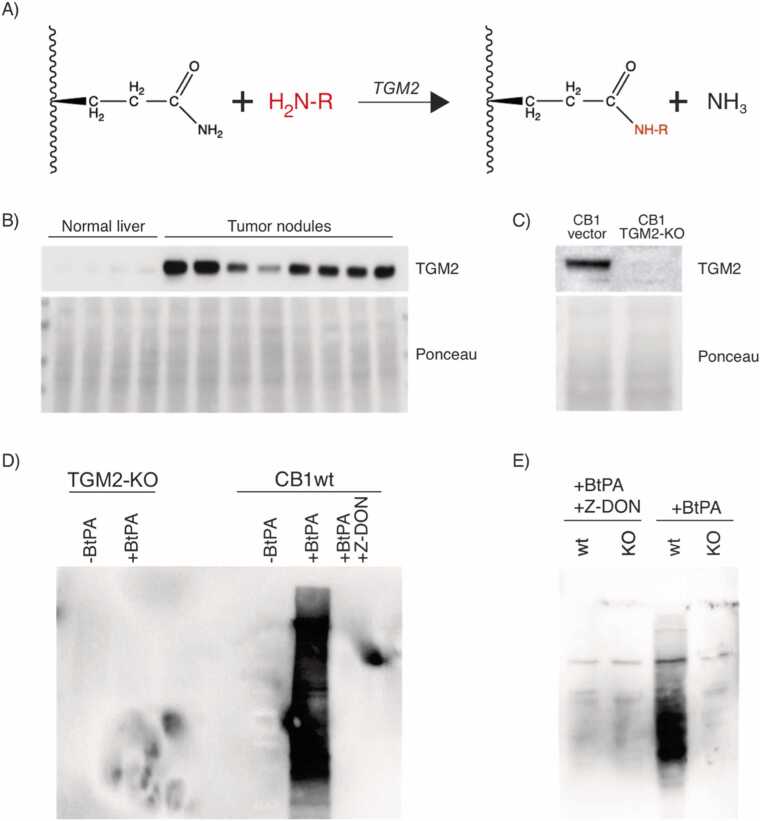
(A) Transamidation of an acceptor glutamine on a target protein by a transglutaminase. If the moiety (-R) on the primary amine is a polyamine, this will result in polyamination of the target protein. (B) Over-expression of TGM2 in liver tumor nodules compared to normal liver tissue. (C) Crispr knock-out of TGM2 in CB1 cells. (D) In vitro polyamination of CB1 cell lysate with biotin-pentylamine (Bt-PA). (E) In vivo Bt-PA incorporation assay by CB1wt and TGM2-KO cells. Blots were probed with HRP conjugated streptavidin (SA-HRP).

Polyamination is a transamidation reaction that covalently couples a polyamine by its amine group to the γ-carboxamide of a glutamine on a recipient protein (Fig. 1A). This is performed by transglutaminase that can attach not just polyamines, but any primary amine donor to an acceptor glutamine (for example, serotonylation or dopaminylation of proteins by TGMs have been described). TGMs are a family of multifunctional enzymes [14] with 7 members. In mammals an eighth protein, FactorXIIIA, also possesses transamidation activity. The most widely expressed transglutaminase isoform, and the best studied, is TGM2.

We developed an inducible liver cancer mouse model by knocking out the tumor suppressors Pten and Tsc1 [13]. This constitutively activates mTORC1 and mTORC2 signalling leading to rapid tumor development. Proteomics of liver tumor nodules revealed over-expression of TGM2 compared to normal liver (Fig. 1B). This agrees with earlier reports on elevated TGM2 levels in Tsc1 and Tsc2 knock-out cells [15]. Metabolic characterization of this tumor model further revealed an increase in polyamine levels compared to normal mouse liver [16]. Observations pointing in the direction of potential interplay between mTOR activity, oncogenic transformation, TGM2 expression and activity have been reported [15], [17]. The increase in polyamine levels was thus interesting as it can serve as a substrate for TGM2 mediated protein polyamination as part of an oncogenic program.

**Fig. 2:**
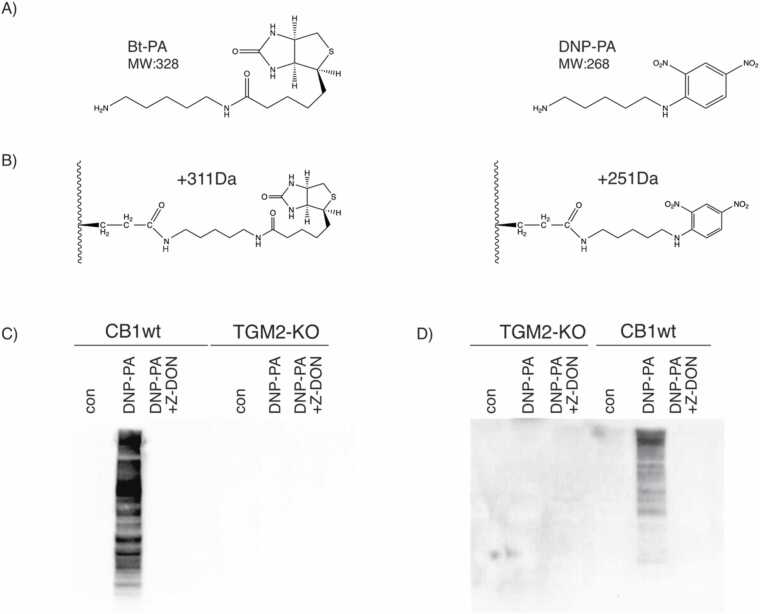
(A) Structures of biotin-pentylamine (Bt-PA) and DNP-pentylamine (DNP-PA). (B) PTM of glutamine with Bt-PA or DNP-PA and the predicted mass shift. (C) In vitro polyamination of CB1 cell lysate with DNP-PA. (D) Incorporation of DNP-PA by CB1 cells and polyamination of proteins in vivo. Blots were probed with anti-DNP antibody.

The immortalized CB1 cell line was derived from liver tumor nodules of this double knockout mouse model [13]. It phenocopied the parent tumor by having elevated TGM2 expression (Fig. 1C). We previously showed that CB1 also retains constitutive mTORC1 and mTORC2 activation. TGM2 was knocked out in CB1 by CRISPR (Fig. 1C), and lysates from wild-type and TGM2-KO cells were assayed for TGM2 dependent polyamination activity using biotin-tagged pentylamine (Bt-PA). Bt-PA has a 5-carbon long polyamine moiety similar to cadaverine (Fig. 2A). Bt-PA was incorporated into endogenous proteins in a wild-type CB1 lysate but not in a lysate from TGM2-KO cells (Fig. 1D). Bt-PA incorporation was completely blocked by the TGM2-specific inhibitor Z-DON. Similar results were obtained with in vivo incorporation and labelling with Bt-PA (Fig. 1E).

Taken together, these results demonstrate that protein polyamination in CB1 relies solely on TGM2. CB1 has elevated TGM2 expression and is derived from tumors with high polyamine levels. Assuming the existence of oncogenic regulatory pathways in which polyamination may perform a signalling function, this would suggest that downstream polyaminated targets of TGM2 would likewise be present in these cells. Taken together, these criteria suggest CB1 is a good model system for detection of TGM2-dependent polyamination.

### 2.2 In vitro polyamination and enrichment of labelled proteins

As polyamination activity is provided solely by TGM2 in CB1, we could directly use whole cell lysates to perform labelling with tagged polyamines using the endogenous TGM2 activity present in the lysate. Two different polyamine tags were used for labelling: Bt-PA and DNP-PA (Fig. 2A). The different sets of reagents and downstream processing for each label helped rule out systematic error in peptide enrichment. Additionally, the different molecular weights of the tags facilitated subsequent detection of predicted mass shifts in the spectra corresponding to the tag employed (Fig. 2B).

We performed labelling with DNP-PA on CB1 using the same conditions as for Bt-PA (Fig. 1) to confirm that the DNP-PA tag can be used for TGM2-specific labelling in vitro and in vivo (Fig. 2C, D).

### 2.3 MS identification of peptides enriched for polyaminated tags

**Fig. 3:**
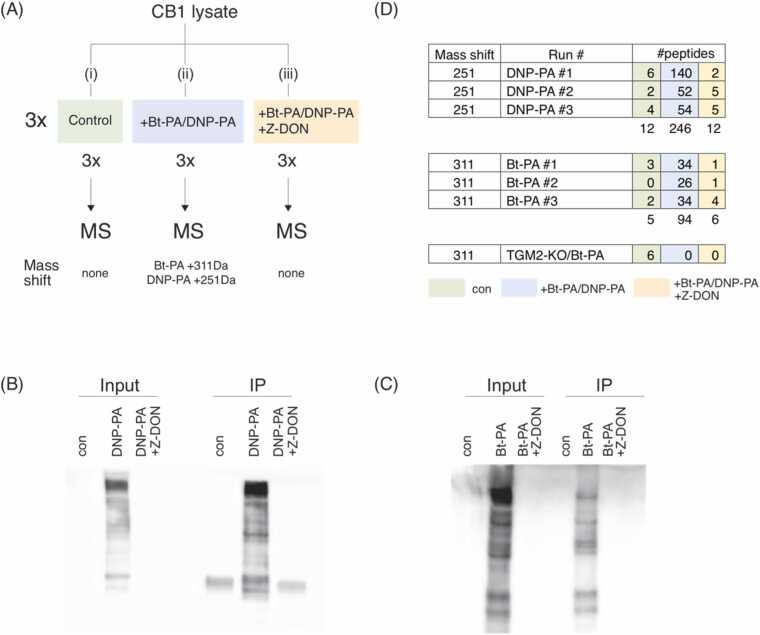
(A) Work-flow for detection of TGM2 polyaminated targets. Peptides bearing expected mass shifts are only expected in the condition with label added and no TGM2 inhibition (blue). (B, C) Examples of quality control performed for each experiment to show labelling and enrichment before sending IP samples for MS. Blots were probed with anti-DNP antibody or SA-HRP. (D) Tabulation of number of peptides detected for the work-flow presented in (A), and for a control experiment using TGM2-KO lysate.

For MS identification of polyaminated peptides, the following work-flow was set up (Fig. 3A). In vitro polyamination reactions of CB1 lysates were performed either (i) without labelled-PA, (ii) with labelled-PA, and (iii) with labelled-PA and Z-DON. This set of reactions was performed independently 3 times making for 3 biological replicates, with each condition performed in triplicate. Under this schema, we expect to detect modified peptides only under condition (ii), with conditions (i) and (iii) serving as internal negative controls. We also performed triplicate reactions using Bt-PA with TGM2-KO lysate as a further negative control of our methodology.

Polyaminated proteins were enriched from the post-reaction mixture by immunoprecipitation with agarose bound anti-DNP and anti-biotin antibody beads (Figs. 3B, 3C). Enrichment of Bt-PA tagged proteins by streptavidin beads gave poor MS results regardless of whether the peptides were liberated by boiling or on-bead digestion, leading us to switch to anti-biotin antibody beads for immunoprecipitation.

Antibody-bound proteins were subjected to on-bead trypsin digestion and eluted peptides processed as described in Methods. Samples were then analyzed by LC-MS/MS.

### 2.4 Identification of peptides with expected mass shifts

Acquired raw-files were analyzed by open search to identify delta mass of either + 251.0906 Da or + 311.16675 Da corresponding to which label was employed (DNP-PA and Bt-PA respectively). Raw data files are deposited at: ftp://MSV000098137@massive-ftp.ucsd.edu. The majority of peptides shifted by the predicted mass values were detected in the expected condition (+tagged-PA), with significantly fewer calls in untagged or TGM2-inhibited conditions (Fig. 3D). Setting stringent conditions, 66 modified sites were detected from 51 proteins (Table 1).

**Table 1:** Combined list of polyaminated peptides identified with the different tagged probes. Acceptor glutamines shown in red. The last two columns indicate in which of the 3 MS runs the peptide was detected. Previously identified acceptor glutamines are referenced in [12], [22], [23], [24], [25]. The Transdab Wiki [22] lists TGM2 polyaminated targets and their primary reference. “As reported” means the acceptor Gln has been previously reported and corresponds with our identification. “Not reported” refers to proteins that have been identified as polyaminated, but without identification of the acceptor Gln which are identified here for the first time. “Diff from reported” indicates that a different Gln was previously identified as polyaminated. “Novel” indicates a protein and its Gln acceptor site identified in this study for the first time.

In vitro labelling of TGM2-KO lysate with Bt-PA did not yield any modified peptides (Fig. 3D). All 6 detections came from the unlabelled control, of which 2 were immunoglobulins used in the post-reaction pull-down. This suggests that these detections are spurious due to the higher sensitivity settings to detect any modified peptide present.

### 2.5 TGM2 polyaminated targets

Identified peptides are curated in Table 1. While many peptides were detected in a single run, many others were picked up in multiple runs. There was a substantial overlap between the glutamine acceptor sites that were identified using the different tags, lending confidence to the approach.

We found a substantial number of novel targets that have not been previously reported as TGM2 polyaminated substrates. We also identified hits previously reported as being polyaminated, but for which the glutamine acceptor was not identified. The convention used to annotate the glutamine acceptor sites are as follows: (i) Novel protein and acceptor Gln detected only in this study, (ii) Not reported, target has been reported to be polyaminated but Gln acceptor was not identified, (iii) As reported, target has been reported with polyamination at the same site, (iv) Different from reported, target has been reported but with a different Gln acceptor from this study.

Many of the targets were structural proteins (Actb/Actg1, Transgelin2, Pdlim1, Centlein, Mapre1, Zyxin, Filamin A, Annexin A1, Tubulin alpha-1a, Tubulin beta-1, Tubulin beta-5). The detected peptides are identical for Actb (Actin, cytoplasmic 1) and Actg1 (Actin, cytoplasmic 2) and we could not distinguish which isoform, or if indeed both, are modified. Targets belonging to the translation machinery were also identified (Eef1a1, Eef1g, Eif4A1, Eif4b, Eif4h, Rps13, Rpl17, Rpl29). Interestingly, the modified glutamines (Q47, Q431) on Eef1a1 are on a similar conserved motif within the protein, suggesting some specificity in their selection. All these targets, including Gapdh and histone H2ac4 are abundant and could indicate an identification bias towards well represented proteins. Of particular interest is polyamination of Grb10 at Q475 as it is flanked by S474 and S476. Grb10 is a negative regulator of mTORC1 that is phosphorylated by mTORC1 at these serine sites in an autoregulatory loop [18], [19]. The juxtaposition of a potential polyaminated site between these 2 regulatory serines may indicate cross-talk between different post-translational modifications. As the CB1 cell line is constitutively active for mTORC1, we investigated if any perturbation of TGM2 or polyamine levels may affect mTORC1 downstream signalling. We either inhibited TGM2 activity with Z-DON, depleted endogenous polyamine pools with APCHA, DFMO or 4-MCHA, or supplemented cells with excess putrescine, spermidine and spermine. Under none of these conditions was any effect seen on a read-out for mTORC1 activity (baseline phosphorylation levels of the mTORC1 substrate S6K, data not shown).

The glycolytic enzyme Gapdh was modified at 3 distinct sites with polyamination on Q202 found in every run. Beyond its essential role in glucose catabolism, Gapdh has many unrelated moonlighting functions and is extensively post-translationally modified [20]. Interestingly, one of the polyaminated glutamines (Q183) lies between a threonine and lysine. These two sites are co-modified by phosphorylation and sumoylation respectively [21]. As with Q475 in Grb10, the proximity of a polyaminated site within a regulatory hotspot may indicate an as yet undescribed role for polyamination in signalling cross-talk.

**Fig. 4:**
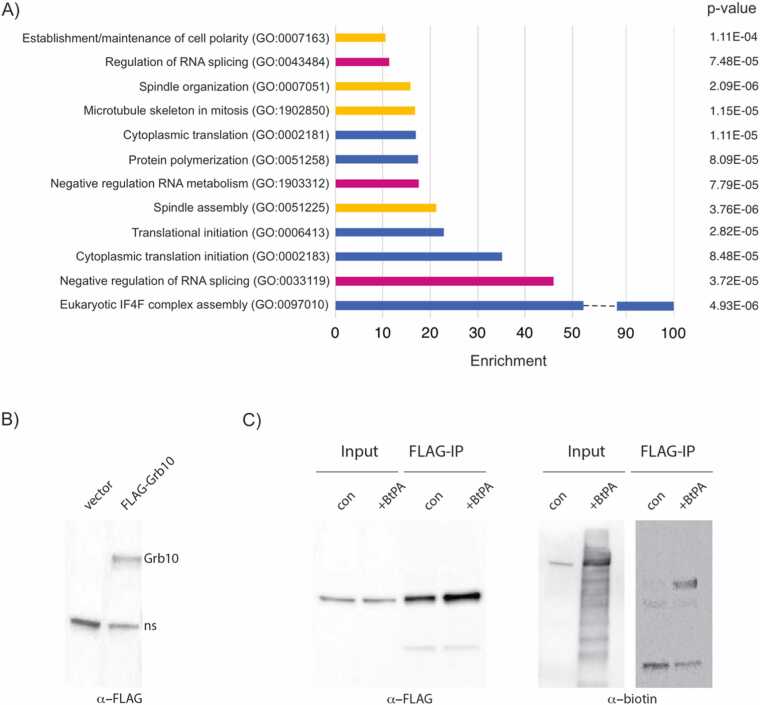
(A) GO analysis enriched biological processes for the identified proteins. Only processes enriched over 10-fold are shown. Processes are loosely grouped as relating to translation (blue), RNA splicing/metabolism (red), and cytoskeletal organization (yellow). Enrichment of “Eukaryotic IF4F complex assembly” was maxed out at 100-fold. Raw p-values shown to the right. (B) Cloning and overexpression of FLAG-Grb10 in CB1. (C) Immunoblots showing FLAG IP of FLAG-Grb10 from control and Bt-PA treated CB1 cell lysate. The same samples were probed for the presence of the biotin tag using an anti-biotin antibody.

A gene ontology analysis was performed. Only processes enriched over 10-fold are shown (Fig. 4A). The most highly represented biological process is protein translation which accurately mirrors the number of translation factors and ribosomal proteins detected. Also significantly enriched is RNA metabolism (splicing and turnover). As with previous studies, we detected various structural proteins (actin, tubulin, filamin, zyxin) which suggest a link to cytoskeletal organization.

### 2.6 Validation of in vivo Grb10 polyamination

As our targets were identified from in vitro polyaminated cell lysate, we sought to validate if they were also polyaminated in vivo. Grb10 was chosen as an example to validate in vivo polyamination. FLAG-tagged Grb10 was over-expressed in CB1. Grb10 is expressed at low levels and the endogenous protein is unlikely to compete with the over-expressed FLAG-construct for the polyamine label (Fig. 4B). In vivo labelling was performed by Bt-PA incorporation into intact cells and FLAG-Grb10 protein was enriched from cell lysates by FLAG purification (Fig. 4C, left panel). FLAG-purified Grb10 was probed for the presence of a Bt-PA tag with an anti-biotin antibody. As shown, a specific signal was detected in the FLAG-Grb10 pulldown (Fig. 4C, right panel) indicating incorporation of Bt-PA into FLAG-Grb10 by in vivo polyamination. Attempts to detect Q475 polyamination in vivo on Grb10 by mass spectrometry were unsuccessful. In vivo polyamination is much less efficient than in vitro polyamination (compare Fig1D vs E, Fig. 2C vs D). Polyaminated peptides are poorly detected by current MS methods and material limitation most likely precluded identification the target peptide.

## 3 Discussion

Our study is the first proteomic survey of TGM2 polyamination since Yu et al. in 2015 [12]. They identified 233 target proteins compared to 51 by us. There was a small overlap of 7 proteins between the two datasets (Filamin A, Histone H2ac4, Khsrp, Mapre1, Tuba1a, Tubb5, Ybx3). Nevertheless, in 2 of these cases, H2ac4 and Mapre1, the Gln-acceptors identified were identical which is highly unlikely to be by chance given the small sample size. The small overlap could be due to cell, tissue or species differences (mouse liver CB1 vs. human cervical carcinoma HeLa), or methodology (their proteins were pulled down using an anti-spermine antibody). There was also an overlap with the Transdab database [22] that lists 156 TGM2 polyaminated targets. These hits were Actb/Actg1, Annexin A1, Eef1a1, Eef1g, Filamin A, Gapdh, and Nucleophosmin 1. Only a few glutamine acceptor sites were identified on Transdab but there was one common hit with the same Gln acceptor site (Annexin A1). We also found several hits in common with an earlier study by Orru et al. (Eef1a1, Eef1g, Fasn, Filamin A), however, there were no Gln-acceptor sites identified in that study [23]. There were hits in common with earlier studies based on organophosphate stimulation of TGM2 in mouse cells. Similar to our methodology, Bt-Cad was incorporated into proteins in stimulated cells and tagged proteins were enriched and analysed by mass spectrometry, however, only enriched proteins were identified and not their acceptor glutamine sites. Seven of our hits (Tubb1, Tubb5, Eef1a1, Eef1g, Gapdh, Nudc, Actg1) are present in their list of 52 enriched proteins from TGM2 stimulated N2a neuroblastoma cells [24]. In a follow up study by the same group using C6 glioma cells, 4 of our hits (Tubb1, Tubb5, Actg1 and Kif5b) were present out of a list of 22 enriched proteins [25]. The overlap between our study (51 proteins) and these other lists, despite the small number of identified proteins involved, lends support to the suggestion that they represent genuine TGM2 polyaminated targets.

Our remaining 39 hits are novel. There is no apparent motif flanking the acceptor-Glns to indicate a specific recognition motif for TGM2 polyamination. A recent study on histone polyamination by TGM2 concluded that an important criterion dictating modification is steric accessibility of the polyaminated site [26]. Our observation that polyamination of Gapdh and Grb10 are at modification hotspots appears to be in line with this notion. A gene ontology analysis of our hits revealed over-representation of proteins involved in translation, RNA metabolism and cytoskeletal organization. A caveat to this analysis is that many of these proteins are highly abundant (cytoskeletal proteins, ribosomal proteins, translation complex components) and this, coupled with the difficulties in peptide identification, may bias the analysis towards abundant proteins that stand a better chance of being detected. An additional point to note is that the overwhelming majority of labelled proteins were not identified by mass spectrometry. Thus, there are potentially other processes involving polyaminated proteins that still remain to be identified.

A limitation of this study is that the polyamine tags employed, Bt-PA and DNP-PA, have a 5-carbon long polyamine chain that make them structurally similar to cadaverine instead of the more physiologically abundant and relevant polyamines (putrescine, spermidine and spermine). Unfortunately, tagged versions of putrescine, spermidine or spermine compatible with enrichment strategies are not commercially available. Other methods of labelling and enriching polyaminated proteins have been described, such as click chemistry [27] and in vitro crosslinking [28]. Having a variety of orthogonal approaches to enrich post-polyaminated proteins provides cross-validation and thus lends confidence.

Nevertheless, the main obstacle appears to be at the level of proteomic analysis and detection of polyaminated peptides. We observed that despite pulling down a large number of tagged proteins (Figs. 3B, 3C), only very few targets were identified by MS, suggesting that there is substantial attrition of material during sample preparation or MS analysis. This is a major hurdle in the proteomic study of polyamination and remains an outstanding issue in the field.

## 4 Material and methods

### 4.1 Cell culture

CB1 cells are immortalized cells derived from a mouse liver cancer tumor [13]. The cells are knocked out for Pten and Tsc1 and have constitutively active mTORC1 and mTORC2 activity. Cells were cultured in in Iscove’s medium supplemented with 10 % fetal bovine serum, 2 mM L-glutamine, penicillin (100 U/ml), and streptomycin (100μg/ml) at 37°C and 5 % CO_2_. FLAG-Grb10 (Origene RC220715L3) and FLAG-GAPDH (Origene RC202309L3) were transfected into CB1 using JetPrime reagent and transfectants selected by puromycin selection (1μg/ml). TGM2-KO cells were obtained by Crispr knockout (Santa Cruz sc-423375).

### 4.2 FLAG-IP

Cell pellets were scraped in ice-cold PBS containing protease and phosphatase inhibitors. Pellets were dounced 30x in 2 ml PBS and cleared by centrifugation at 20,000 g, 20 min, 4°.

50μl of washed anti-FLAG beads was added to 500μg cleared lysate (at 1μg/μl concentration) and rotated for 4–6 h at 4°. Beads were washed 3x1ml ice-cold PBS and bound proteins eluted with Flag peptide (2 ×30μl, 0.5μg/ml).

### 4.3 Immunoblotting

Cells pellets were lysed in M-PER buffer (ThermoFisher) and total protein (20–40 μg) resolved on SDS-PAGE gels. Proteins were blotted onto nitrocellulose membranes and probed with anti-DNP (SantaCruz sc-69698) or FLAG antibodies (Sigma Anti-FLAG M2 F1365). Biotin was probed with an HRP-Streptavidin conjugate (Sigma RABHRP3). TGM2 was probed with TGase2 Antibody (4G3), SantaCruz sc-73612.

### 4.4 Polyamination reactions

In vitro: CB1 cell whole cell lysates were made using M-PER extraction buffer containing protease inhibitors (cOmplete, Roche, 0.5 mM PMSF) and phosphatase inhibitors (PhosSTOP, Roche). 100μg of WCL lysate was diluted in 50 mM MOPS, 5 mM CaCl_2_ and 1 mM Biotin-pentylamine (Bt-PA, Sigma) or DNP-pentylamine (DNP-PA, Zedira) was added. The reaction was incubated at 37° for 3 h. 100μM Z-DON was added for negative controls. At the end of the reaction, reaction products were passed through a size exclusion column (3 kDa cut-off) to remove unincorporated Bt-PA and DNP-PA.

In vivo: Confluent CB1 cells were directly treated with 1μM ionomycin and 1 mM labelled polyamines Bt-PA or DNP-PA. Cells were incubated for 4 h before harvesting by scraping. Downstream processing of cell lysates was similar as for in vitro samples.

### 4.5 Enrichment of polyaminated proteins

Post-reaction, cell lysates were diluted to 800∂μl in 0.3 % CHAPS buffer and anti-biotin (SantaCruz sc-101339AC) or anti-DNP (SantaCruz sc-69698AC) beads were added. Tubes were rotated for 4–6 h at 4°. Beads were washed 3x1ml in ice-cold 0.3 % CHAPS buffer and bound proteins either eluted by boiling for immunoblotting, or trypsin digested off beads for MS.

### 4.6 Tryptic digest and LC MS/MS analysis

Affinity purified samples were subjected to on-bead digestion (adapted from PMID: 20479470). Resin was washed three times with detergent free wash solution and collected by centrifugation. Proteins were eluted by incubation in 1.6 M Urea, 100 mM Ammonium bicarbonate, 5 μg/ml trypsin, pH 8 for 30 min at 27°C shaking at 1200 rpm followed by two incubations in 1.6 M Urea, 100 mM Ammonium bicarbonate, 1 mM TCEP, pH 8. After each incubation, resin was collected by centrifugation and supernatants were collected and pooled. TCEP and Chloroacetamide were added at a final concentration of 10 mM and 15 mM, respectively, and samples were incubated for 1 h at 37°C shaking at 600 rpm prior to the addition of 0.5 μg trypsin and incubation for 12 h at 37°C shaking at 300 rpm. Tryptic digest was acidified (pH<3) using TFA and desalted using C18 reversed phase spin columns (Microspin, Harvard Apparatus) according to the manufacturer’s instructions. Peptides were dried under vacuum and stored at −20°C.

Dried peptides were resuspended in 0.1 % aqueous formic acid and subjected to LC–MS/MS analysis using a Q Exactive Plus Mass Spectrometer fitted with an EASY-nLC 1000 (both Thermo Fisher Scientific) and a custom-made column heater set to 60°C. Peptides were trapped on a PepMap Neo Trap Cartridge (Thermo Fisher Scientific) and resolved using a RP-HPLC column (75um × 30 cm) packed in-house with C18 resin (ReproSil-Pur C18–AQ, 1.9 μm resin; Dr. Maisch GmbH) at a flow rate of 0.2 μl/min. The following gradient was used for peptide separation: from 5 % B to 10 % B over 5 min to 35 % B over 45 min to 50 % B over 10 min to 95 % B over 2 min followed by 18 min at 95 % B. Buffer A was 0.1 % formic acid in water and buffer B was 80 % acetonitrile, 0.1 % formic acid in water.

The mass spectrometer was operated in DDA mode with a total cycle time of approximately 1 s. Each MS1 scan was followed by high-collision-dissociation (HCD) of the 10 most abundant precursor ions with dynamic exclusion set to 45 s. MS1 scans were acquired in Profile mode with a scan range from 350 to 1600 *m/z*, AGC target set to 3e6, a maximum injection time of 100 ms and a resolution of 70000 FWHM (at 200 *m/z*). MS2 scans were acquired in Centroid mode with a scan range from 200 to 2000 *m/z*, AGC target set to 1e5, a maximum injection time of 100 ms and a resolution of 35000 FWHM (at 200 *m/z*). Singly charged ions and ions with unassigned charge state were excluded from triggering MS2 events. The normalized collision energy was set to 27 %, the mass isolation window was set to 1.4 *m/z* and one microscan was acquired for each spectrum.

The acquired raw-files were searched using MSFragger (v. 3.7) implemented in FragPipe (v. 19.1) against a Mus musculus database (consisting of 17085 protein sequences downloaded from Uniprot on 20220222) and 392 commonly observed contaminants. The default LFQ-MBR workflow was used. with minor modifications: In the MSFragger Spectral Processing tab, “Require precursor” was unchecked, MSBooster was disabled, in the Quant (MS1) tab “MBR top runs” was set to 100,000, “Top N ions” was set to 3 and “Min freq” was set to 0.5. A variable modification on Q with a delta mass of either 251.0906 Da (DNP-PA) or 311.16675 Da (Bt-PA) was added.

### 4.7 Gene ontology analysis

GO analysis was performed for the list of proteins in Table 1 using PANTHER 19.0 from the Gene Ontology Resource [29], [30]. False discovery rate was set at p < 0.05 and only processes with a greater than 10-fold enrichment were included.

## Ethics statement

This research presents an accurate account of the work performed, all data presented are accurate and methodologies detailed enough to permit others to replicate the work.

This manuscript represents entirely original works and or if work and/or words of others have been used, that this has been appropriately cited or quoted and permission has been obtained where necessary.

This material has not been published in whole or in part elsewhere.

The manuscript is not currently being considered for publication in another journal.

That generative AI and AI-assisted technologies have not been utilized in the writing process or if used, disclosed in the manuscript the use of AI and AI-assisted technologies and a statement will appear in the published work.

That generative AI and AI-assisted technologies have not been used to create or alter images unless specifically used as part of the research design where such use must be described in a reproducible manner in the methods section.

All authors have been personally and actively involved in substantive work leading to the manuscript and will hold themselves jointly and individually responsible for its content.

## Author statement

**DB** conceived and performed experiments and wrote the manuscript. **DR** developed and performed proteomic analysis, **SS** and **SP** developed cell lines, **MNH** provided input and wrote the manuscript.

## CRediT authorship contribution statement

**Sunil Shetty:** Resources. **Sujin Park:** Resources. **Hall Michael N:** Writing – review & editing, Supervision. **Don Benjamin:** Writing – review & editing, Writing – original draft, Validation, Project administration, Methodology, Investigation, Conceptualization. **Danilo Ritz:** Methodology, Formal analysis, Data curation.

## Declaration of Competing Interest

We declare no conflict of interest.
